# When Blood Leaves a Trace: Diagnosing Superficial Siderosis in a Geriatric Patient

**DOI:** 10.7759/cureus.89025

**Published:** 2025-07-29

**Authors:** Jerin G Viju, Parameswaran Krishnan, Anand M

**Affiliations:** 1 Neurology, Indo American Brain and Spine Center, Vaikom, IND; 2 Neuroradiology, Indo American Brain and Spine Center, Vaikom, IND

**Keywords:** deferiprone, gradient recalled echo (gre), neuro mri, progressive ataxia, sensorineural (sn) hearing loss, superficial siderosis, susceptibility weighted imaging ( swi )

## Abstract

Superficial siderosis (SS) is a rare neurodegenerative condition caused by chronic or recurrent bleeding into the subarachnoid space, leading to the deposition of hemosiderin in the subpial layers of the brain, brainstem, spinal cord, or cranial nerves. SS is categorized into two main forms based on its anatomical distribution: cortical superficial siderosis (cSS) and infratentorial superficial siderosis (iSS). Among these, iSS is further subdivided into Type 1 (classical iSS) and Type 2 (secondary iSS). Classical iSS refers to cases in which there is no radiologically confirmed single spontaneous or traumatic intracranial hemorrhage and is typically associated with chronic bleeding sources, such as cranial or spinal dural defects. In contrast, secondary iSS is characterized by the presence of a radiologically identifiable single intracranial hemorrhagic event, either spontaneous or traumatic, which is considered the likely cause of the siderosis. Patients typically present with SS in the fourth to sixth decades of life. The prevalence of SS has been increasing in recent years due to the more widespread use of magnetic resonance imaging (MRI). Early imaging and diagnosis are crucial for identifying the bleeding source and enabling timely intervention, which may prevent further neurological deterioration. We report the case of a 69-year-old woman with a history of subdural hematoma and surgical intervention who presented with progressive bilateral hearing loss, imbalance, headache, and hypogeusia. MRI of the brain revealed classical imaging features of SS. Although further evaluation was recommended, the patient declined extensive diagnostic work-up. Empirical treatment with the iron-chelating agent deferiprone was initiated. This case highlights the diagnostic challenges of SS and emphasizes the importance of comprehensive imaging and early intervention.

## Introduction

Superficial siderosis (SS) is characterized by the chronic deposition of iron and hemosiderin in the pial and subpial regions of the central nervous system, resulting from recurrent or intermittent bleeding into the subarachnoid space [1]. The most common etiology is dural pathology, where chronic bleeding from fragile blood vessels around a dural tear is suspected to lead to hemosiderin deposition [2]. Classical symptoms include progressive gait ataxia, sensorineural hearing loss, and myelopathy [3].

SS is broadly categorized into two anatomical subtypes: cortical SS (cSS) and infratentorial SS (iSS). Cortical SS is typically associated with cerebral amyloid angiopathy (CAA) in older adults and is characterized by hemosiderin deposition in the cerebral cortices, particularly the convexities. In contrast, iSS affects infratentorial structures such as the brainstem, cerebellum, and spinal cord, and is more commonly linked to chronic bleeding from spinal dural defects [4].

A classification system has been proposed that divides iSS into Type 1 (classical) and Type 2 (secondary) forms. Type 1 iSS is defined by the absence of a single radiologically confirmed hemorrhagic event and is often associated with progressive neurological symptoms such as sensorineural hearing loss, gait ataxia, and myelopathy. In contrast, Type 2 iSS follows a known, single traumatic or spontaneous intracranial hemorrhage and is less frequently associated with the classical triad [5].

Epidemiologically, SS has historically been underdiagnosed due to its insidious onset and nonspecific presentation. However, with the widespread use of blood-sensitive MRI sequences such as susceptibility-weighted imaging (SWI) and gradient echo (GRE), detection rates have increased [4]. Despite this, there are no universally accepted diagnostic criteria for SS, though diagnosis is generally made radiologically based on characteristic hypointense linear signals on iron-sensitive MRI sequences [5].

We report a case of SS in a 69-year-old woman with a prior history of subdural hematoma, emphasizing its clinical presentation, radiological diagnosis, and the challenges of management.

Geriatric patients present a unique diagnostic challenge in SS due to the insidious onset and non-specific nature of its symptoms, which often overlap with common age-related conditions such as presbycusis, degenerative spine disease, and gait instability. These features can lead to misattribution and delayed diagnosis. Furthermore, coexisting comorbidities and reduced access to specialized imaging in this population may obscure the clinical picture. As such, a high index of suspicion and the use of appropriate blood-sensitive MRI sequences are crucial for timely recognition in older adults.

## Case presentation

A 69-year-old right-handed woman presented to the neurology outpatient department with a four-month history of gradually progressive bilateral hearing impairment, initially manifesting as a sensation of fullness and reduced hearing acuity in the right ear, followed by similar symptoms on the left side. The hearing loss was insidious in onset and non-fluctuating. Over the following weeks, she developed a persistent headache, generalized slowness, unsteadiness while walking, and a noticeable reduction in her sense of taste (hypogeusia), which were noted following a recent febrile illness. There was no history of nausea, vomiting, photophobia, or focal limb weakness.

Her past medical history included well-controlled hypertension and a subacute left frontoparietal subdural hematoma, for which she underwent burr-hole evacuation approximately three years ago. She had no history of anticoagulant use, vascular malformations, or spinal procedures.

Neurological examination revealed an alert and oriented patient. Cranial nerve assessment demonstrated bilateral sensorineural hearing loss, more pronounced on the right; confirmed by Rinne and Weber testing, with intact tympanic membranes. Taste sensation was diminished on the anterior two-thirds of the tongue. Cerebellar examination showed a broad-based, unsteady gait with a positive Romberg sign and mild bilateral dysmetria on finger-nose testing, indicating cerebellar involvement.

Given the constellation of bilateral asymmetric sensorineural hearing loss, cerebellar ataxia, hypogeusia, and a history of prior neurosurgical intervention, SS of the central nervous system was suspected. Audiometry confirmed bilateral sensorineural hearing loss. Routine blood tests, including complete blood count, renal and liver function tests, and inflammatory markers, were unremarkable, effectively excluding infectious and inflammatory causes.

MRI of the brain with SWI revealed blooming susceptibility along the cerebellar folia and pial surface of the brainstem, consistent with Type 1 classical SS. Additional susceptibility blooming was seen along the superior surface of the cerebellar folia, medial aspect of the vermis (as T2 hypointensity), and asymmetrically in the left greater than right Sylvian fissure and the right calcarine fissure. Symmetrical hyperintensity of the bilateral globus pallidi was noted but deemed unrelated. There was no evidence of cerebellopontine angle (CPA) tumors. magnetic resonance angiography (MRA) and magnetic resonance venography (MRV) of the brain and neck were normal, showing no aneurysms, arteriovenous malformations (AVMs), dural arteriovenous fistulae (dAVF), or significant carotid artery stenosis.

Although spinal MRI was recommended to evaluate for potential sources of chronic bleeding, such as a cerebrospinal fluid (CSF) leak or intraspinal tumors (e.g., ependymomas or schwannomas), the patient declined further imaging. Differential diagnoses considered included CPA tumors, spinal neoplasms, chronic hemorrhage from occult vascular malformations, cerebral amyloid angiopathy, and high-grade intracranial stenosis. However, the absence of mass lesions, demyelinating pathology, or acute infarcts, combined with the characteristic imaging findings and clinical picture, supported a working diagnosis of SS.

In the absence of a surgically correctable lesion, and based on the clinical and radiological diagnosis of SS, she was initiated on oral deferiprone. Deferiprone is a lipid-soluble iron chelator capable of crossing the blood-brain barrier and, at a dose of 30 mg/kg/day, has demonstrated the ability to reduce hemosiderin deposition in patients with SS, as shown in preliminary clinical studies and observational trials [6]. Given the patient’s age and comorbidities, a conservative dosing regimen of 500 mg twice daily was selected. Although lower than the commonly studied dose, it falls within the range used in off-label clinical practice.

MRI brain

**Figure 1:**
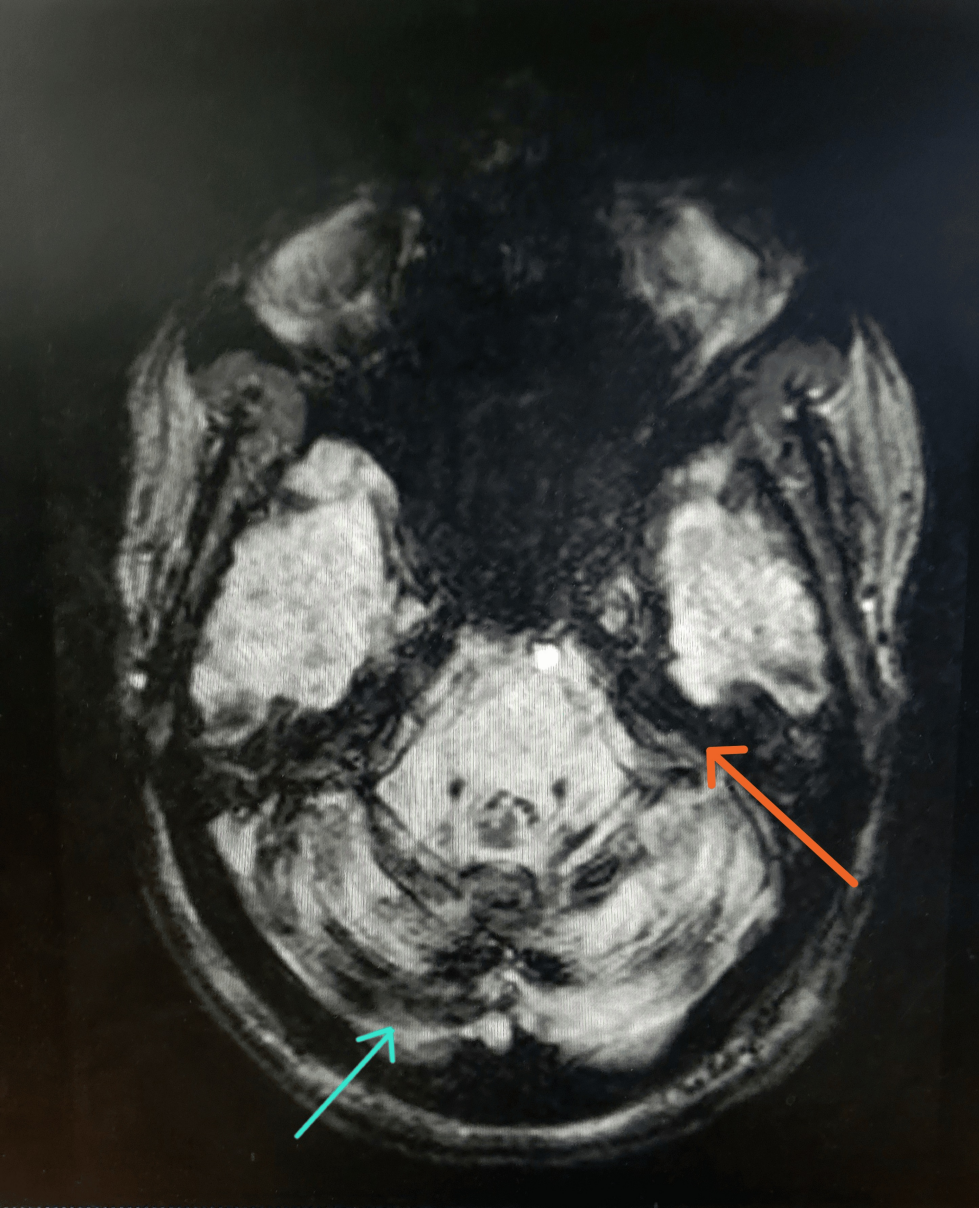
SWI axial image showing iSS Type 1 (classical) SWI axial image through the posterior fossa shows blooming susceptibility along the cerebellar folia (blue arrow) and the pial surface of the brainstem (orange arrow). iSS: infratentorial superficial siderosis; SWI: susceptibility-weighted imaging

**Figure 2:**
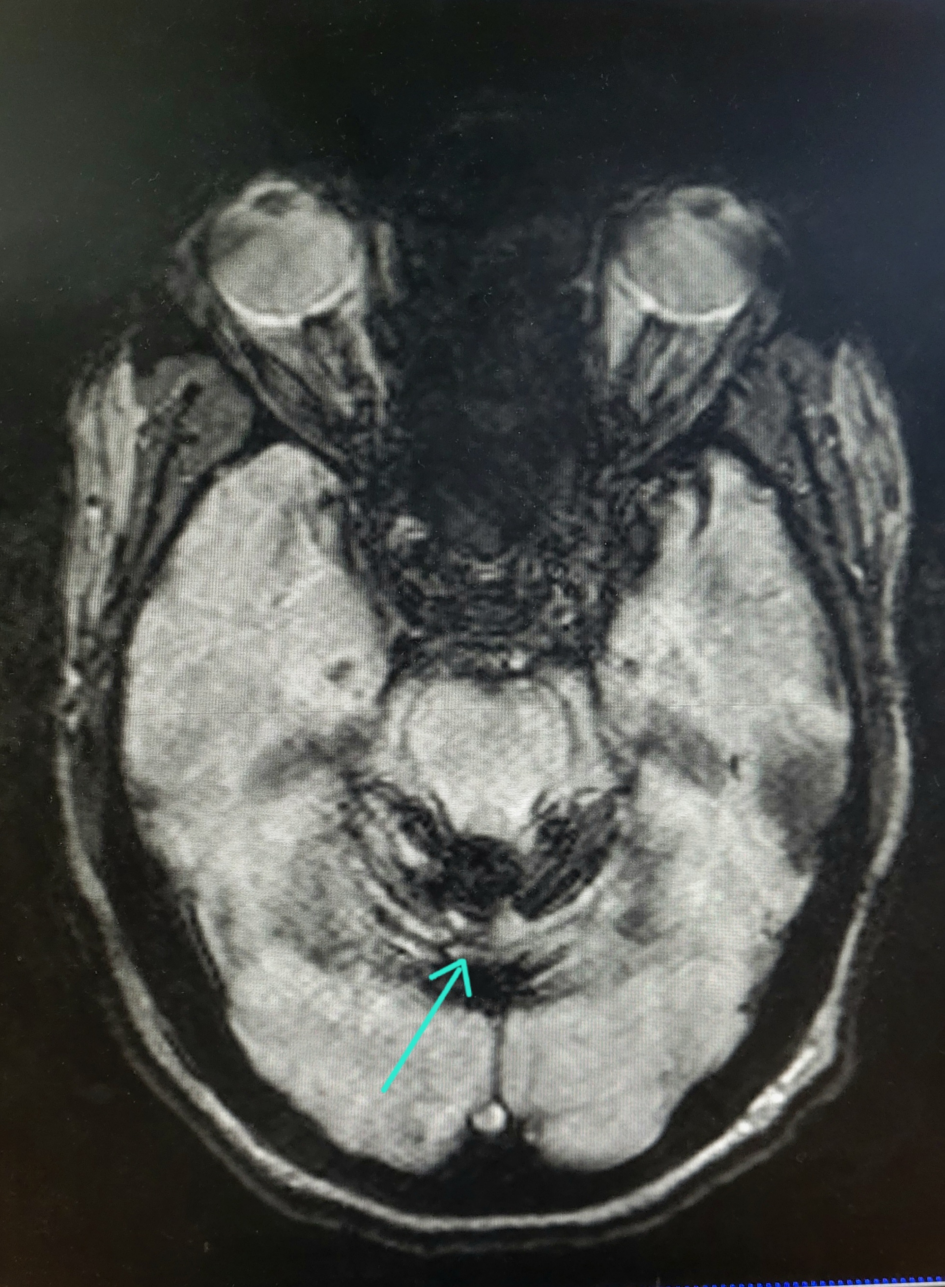
SWI cranial section showing Type 1 iSS Cranial section SWI shows susceptibility blooming along the superior part of the cerebellar folia. iSS: infratentorial superficial siderosis; SWI: susceptibility-weighted imaging

**Figure 3:**
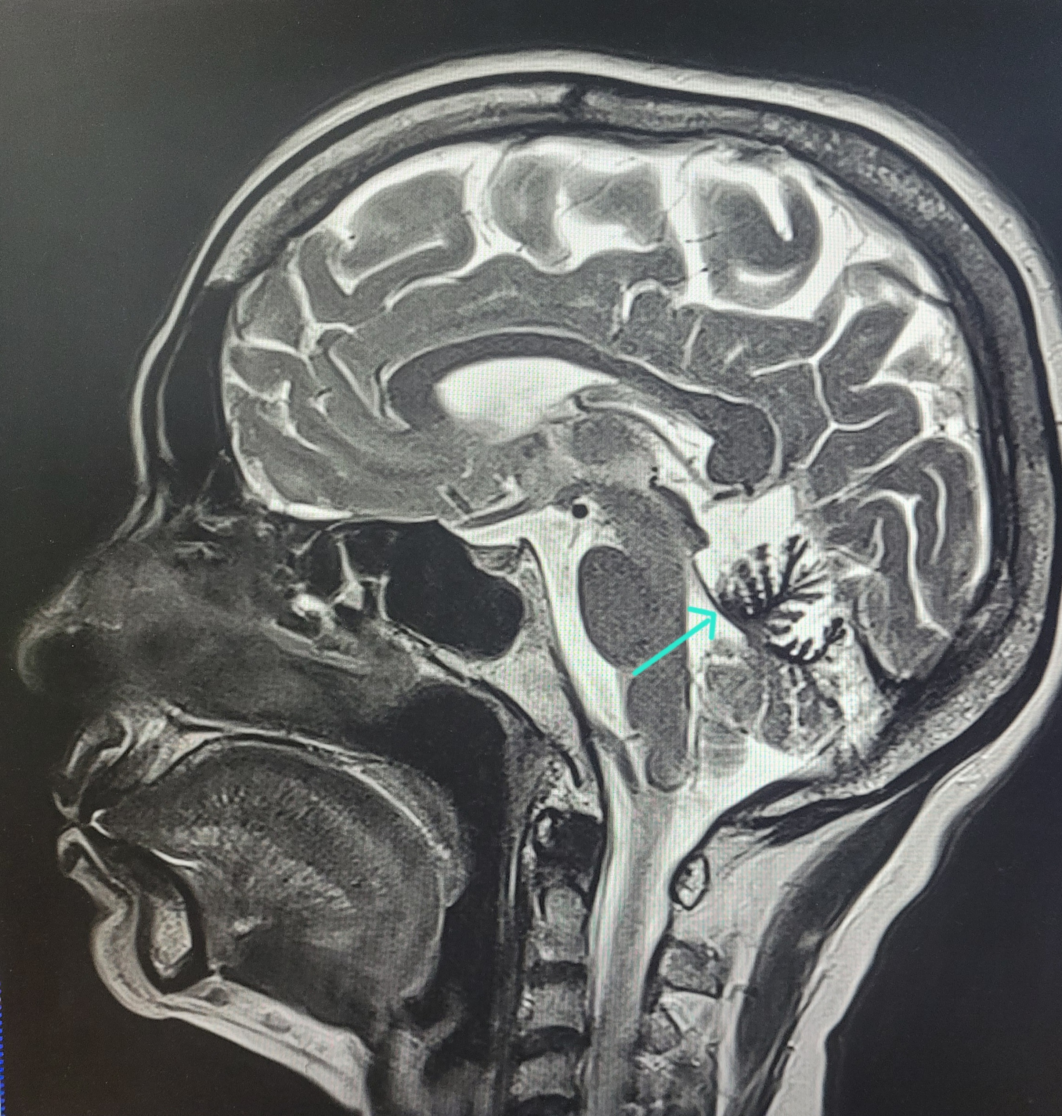
Mid-sagittal MRI showing iSS Type 1 T2-weighted mid-sagittal section MRI showing hypointensity along the medial aspect of vermis. iSS: infratentorial superficial siderosis; SWI: susceptibility-weighted imaging

SWI axial imaging through the posterior fossa demonstrated blooming susceptibility along the cerebellar folia and the pial surface of the brainstem, consistent with Type 1 classical SS. Additional susceptibility blooming was noted along the superior surface of the cerebellar folia and the pial surface of the brainstem. T2 mid-sagittal imaging revealed T2 hypointensity along the medial aspect of the vermis (Figures 1-3).

**Figure 4:**
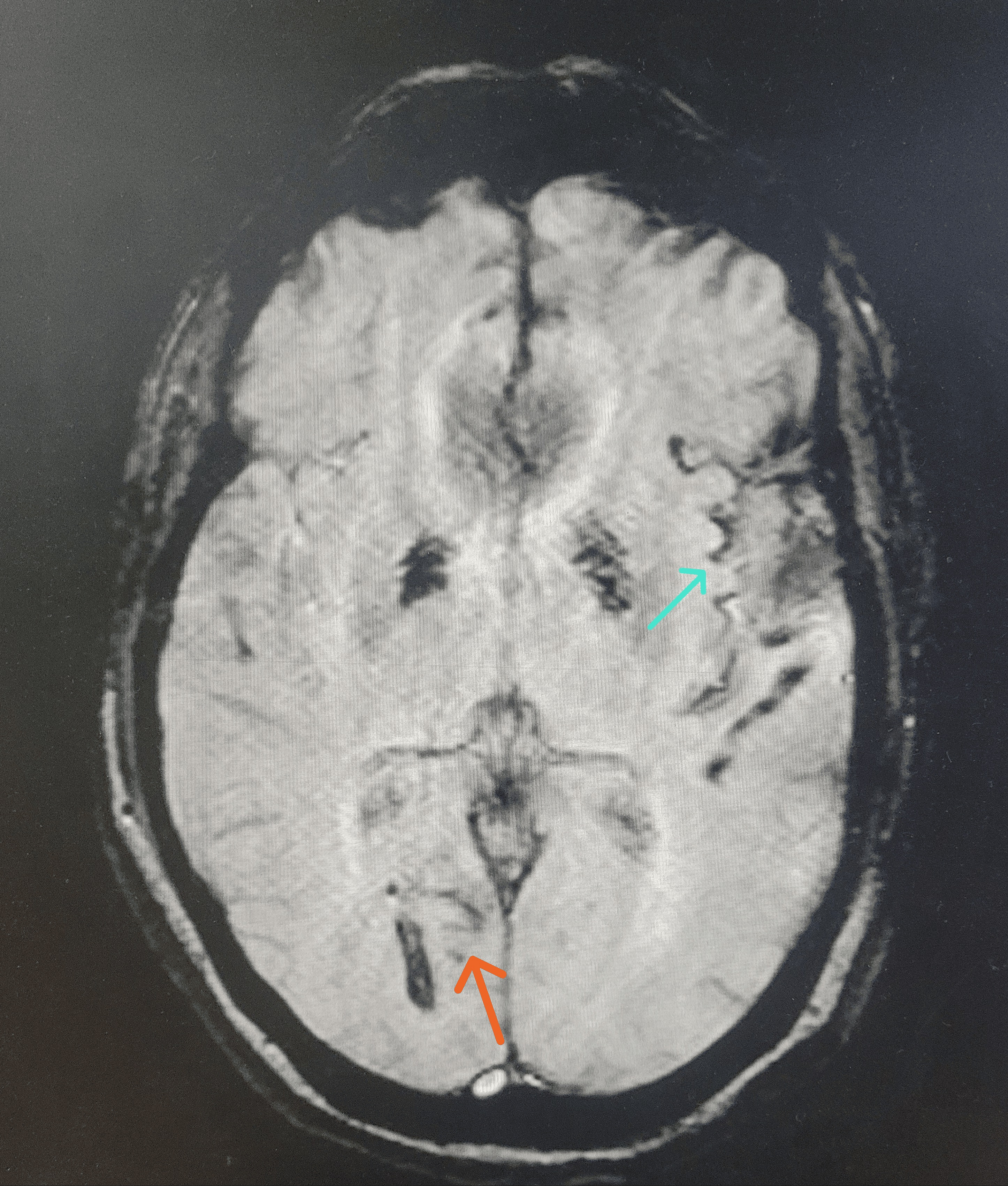
Axial SWI showing supratentorial SS Axial SWI at the level of the basal ganglia shows susceptibility blooming seen asymmetrically in the left greater than right Sylvian fissure (blue arrow) and in the right calcarine fissure (orange arrow). SS: superficial siderosis; SWI: susceptibility-weighted imaging

Axial SWI through the level of the basal ganglia showed susceptibility blooming asymmetrically in the left greater than right Sylvian fissure and in the right calcarine fissure. Symmetrical hyperintensity of the bilateral globus pallidi was observed but considered unrelated (Figure 4).

## Discussion

SS of the central nervous system is a rare, progressive neurodegenerative condition caused by recurrent or chronic bleeding into the subarachnoid space, resulting in hemosiderin deposition on the surface of the brain, brainstem, spinal cord, and cranial nerves. It is frequently insidious in onset and may occur without a clear history of subarachnoid hemorrhage, though prior trauma or intradural surgery is commonly implicated [4]. Our case aligns with these classical features, particularly progressive bilateral sensorineural hearing loss and truncal ataxia. The possibility of an accidental dural breach during subdural hematoma evacuation further highlights the diagnostic challenge posed by its gradual onset and nonspecific early symptoms.

Depending on the location of iron deposition, superficial siderosis can be classified as supratentorial or infratentorial, with the latter being more common. The typical etiology of infratentorial iSS involves spinal dural defects, particularly dural tears, which allow chronic low-volume hemorrhage from fragile bridging veins. Associated intracranial hypotension may exacerbate the process by causing venous engorgement and traction on cerebellar veins. In contrast, cSS (supratentorial) is usually due to leptomeningeal bleeding secondary to conditions such as cerebral amyloid angiopathy [4].

Hemosiderin accumulation is a result of red blood cell breakdown in the CSF. Hemoglobin is degraded into heme, which is further metabolized by neuroglial cells into free iron via heme oxygenase. Iron is then sequestered into ferritin and ultimately hemosiderin. Persistent bleeding may overwhelm this protective mechanism, resulting in iron-mediated free radical damage, lipid peroxidation, gliosis, demyelination, and neuronal loss. Notably, the olfactory nerve appears to be spared [4].

The cerebellum is especially susceptible to siderosis due to several factors: its rich population of Bergmann glia, which upregulate ferritin synthesis; its proximity to the fourth ventricle; and gravitational pooling of blood in the superior cerebellar cistern, particularly in the supine position. Clinically, this preferential involvement often presents with cerebellar ataxia. Sensorineural hearing loss, another hallmark feature, is attributed to prolonged CSF exposure of the glial-rich vestibulocochlear nerve within the pontine cistern [4].

MRI with iron-sensitive sequences such as GRE (gradient-recalled echo) or SWI (susceptibility-weighted imaging) remains the gold standard for diagnosis. Full neuraxis imaging, including brain and spinal MRI, is essential to evaluate the extent of hemosiderin deposition and identify the bleeding source. However, as seen in our case, this evaluation is sometimes limited by patient choice or availability [3]. According to Kumar et al., such limitations are a common challenge in real-world clinical settings and may hinder definitive treatment [2].

In our patient, classical features including bilateral asymmetric sensorineural hearing loss, cerebellar ataxia, and hypogeusia prompted further imaging, which confirmed superficial siderosis. However, efforts to identify the bleeding source were curtailed due to the patient's decision to decline further investigation. Empirical treatment with deferiprone, an iron-chelating agent capable of penetrating the blood-brain barrier, was initiated. Though evidence remains limited, deferiprone may help mitigate hemosiderin accumulation and potentially slow neurological decline in affected individuals [6].

Differential diagnoses

In evaluating this case, several differential diagnoses were considered. A spinal CSF leak or spinal tumor, such as a schwannoma or ependymoma, was initially suspected, given their known association with superficial siderosis through chronic bleeding into the subarachnoid space [7,8]. Chronic bleeding from a dural arteriovenous fistula was also considered, as these lesions can cause insidious hemorrhage and hemosiderin deposition over time [9]. Cerebral amyloid angiopathy was another possibility, particularly in the elderly population, due to its propensity to cause recurrent cortical hemorrhages. Chronic rebleeding from a prior burr hole site was evaluated given the patient's surgical history, which could serve as a persistent source of bleeding [4]. Lastly, intracranial high-grade stenosis with hemorrhagic transformation was considered, especially in the context of vascular risk factors and prior ischemic events [10].

## Conclusions

This case underscores the importance of maintaining a high index of suspicion for SS in patients presenting with progressive bilateral sensorineural hearing loss, cerebellar ataxia, and a history of central nervous system trauma or neurosurgical intervention. Early recognition is critical, as timely imaging and appropriate management may help arrest or slow disease progression. MRI of the entire neuroaxis, utilizing iron-sensitive sequences such as SWI or GRE, remains essential for diagnosis. Comprehensive imaging not only confirms the presence of hemosiderin deposition but also assists in localizing the underlying source of chronic subarachnoid hemorrhage. In the absence of a surgically correctable lesion or when further evaluation is limited, iron chelation therapy with agents like deferiprone may be considered to reduce the iron burden and mitigate neurological decline. Future research should focus on optimizing treatment protocols, particularly in elderly patients, and developing evidence-based guidelines for long-term management and monitoring of this rare but progressive condition.
